# Effect of Light and *p*-Coumaric Acid on the Growth and Expression of Genes Related to Oxidative Stress in *Brettanomyces bruxellensis* LAMAP2480

**DOI:** 10.3389/fmicb.2021.747868

**Published:** 2021-11-25

**Authors:** Daniela Catrileo, Sandra Moreira, María Angélica Ganga, Liliana Godoy

**Affiliations:** ^1^Departamento de Fruticultura y Enología, Facultad de Agronomía e Ingeniería Forestal, Pontificia Universidad Católica de Chile, Santiago, Chile; ^2^Departamento de Ciencia y Tecnología de los Alimentos, Facultad Tecnológica, Universidad de Santiago de Chile, Santiago, Chile

**Keywords:** *B. bruxellensis*, light intensity, oxidative stress, ROS, *p*-coumaric acid

## Abstract

*Brettanomyces bruxellensis* is considered the most significant contaminant yeast in the wine industry since it causes a deterioration in the organoleptic properties of the wine and significant economic losses. This deterioration is due to the production of volatile phenols from hydroxycinnamic acids. These compounds possess antimicrobial properties; however, *B. bruxellensis* can resist this effect because it metabolizes them into less toxic ones. Recent studies have reported that *B. bruxellensis* grows under different stress conditions, including *p*-coumaric acid (*p*CA) but effective methods for its control have not been found yet. Since that in other yeasts, such as *Saccharomyces cerevisiae*, it has been described that light affects its growth, and we evaluated whether the light would have a similar effect on *B. bruxellensis*. The results show that at light intensities of 2,500 and 4,000 lux in the absence of *p*CA, *B. bruxellensis* LAMAP2480 does not grow in the culture medium; however, when the medium contains this acid, the yeast adapts to both factors of stress managing to grow. The expression of genes related to oxidative stress in *B. bruxellensis* LAMAP2480, such as *SOD1*, *GCN4*, and *ESBP6*, showed a higher relative expression when the yeast was exposed to 2,500 lux compared to 4,000 lux, agreeing with the growth curves. This suggests that a higher expression of the genes studied would be related to stress-protective effects by *p*CA.

## Introduction

*Brettanomyces bruxellensis* has been described as the main contaminating yeast during the winemaking process due to its ability to metabolize hydroxycinnamic acids (HCAs; *p*-coumaric acid (*p*CA), ferulic acid, and caffeic acid) in less toxic compounds, such as volatile phenols (Chatonnet et al., 1995; Fleet, 2003; Suárez et al., 2007; Wedral et al., 2010). This is important because HCAs have antimicrobial activity which would be metabolized by this yeast. These give off-favors to the wine, damaging its organoleptic properties (Loureiro, 2003; Suárez et al., 2007) and causing rejection by the consumer along with significant economic losses for the industry (Loureiro, 2003; Suárez et al., 2007; Oelofse et al., 2008; Wedral et al., 2010). Godoy et al. (2016) reported a comparative analysis of the transcriptome and genome profile of the strain *B. bruxellensis* LAMAP2480 grown in the presence of *p*CA, and an early resistance mechanism to this acid was observed, causing generalized stress in the cell, and therefore inducing the expression of genes that encode proton pumps and mechanisms related to the release of toxic compounds.

On the other hand, the presence of this acid activates the expression of different genes related to this response, such as *SOD1*, *ESBP6*, *GCN4*, and *HSP12* (Godoy et al., 2016). Similar results have been described for *Saccharomyces cerevisiae*, where the presence of *p*CA also causes an increase in reactive oxygen species (ROS) and consequently an increase in the expression of genes related to oxidative stress (Piper, 1999; de Nobel et al., 2001; Mascarenhas et al., 2008; Welker et al., 2010; Sugiyama et al., 2016). *SOD1* encodes a cytosolic superoxide dismutase, which is responsible for eliminating radicals produced by the cell and toxic to it (Steinman, 1980; Jamieson, 1998). Furthermore, it has been shown that this superoxide dismutase would be part of a resistance mechanism in the presence of sorbic acid (Piper, 1999; de Nobel et al., 2001). Another gene described is *ESBP6*, which codes for a protein similar to a monocarboxylate permease that promotes the exit of weak acids from the cell, allowing the regulation of intracellular pH and reducing stress levels (Sugiyama et al., 2016; Pereira et al., 2020). Likewise, it has been identified that *GCN4* gene, which codes for a basic leucine zipper domain (b-ZIP) transcription factor and is related to a protection mechanism, inhibits the growth of mutants lacking this gene, and shows overexpression in the presence of hydrogen peroxide (H_2_O_2_; Jamieson, 1998; Mascarenhas et al., 2008). In addition, *HSP12* gene encodes a membrane protein whose function is to stabilize it under stress conditions, including oxidative stress. This protein has been shown to have a protective role when *S. cerevisiae* is exposed to sorbic acid (de Nobel et al., 2001). In the case of mutant strains lacking *HSP12*, an inhibition in their growth was observed (Welker et al., 2010).

The effect of light on microorganisms has generated great interest for further research, considering that it can result in the control of cellular functions, which could not be achieved with diffusion processes (Kusen et al., 2017). In addition, it would allow the activation of biological processes in a non-invasive way. Therefore, studies have been focusing on knowing how this exogenous factor may trigger the expression of genes involved in metabolic pathways of cellular importance (Binder et al., 2016). Thus, for example, it has been described that visible light is harmful to cells, affecting cellular respiration by destroying cytochromes (Epel and Butler, 1969; Ninnemann et al., 1970; Woodward et al., 1978; Ułaszewski et al., 1979) or by the production of ROS, which can react with biomolecules, such as lipids, nucleic acids, and proteins and inactivate their function (Toledano et al., 2003; Perrone et al., 2008). This causes oxidative stress in the cell, as does the presence of *p*CA (Piper, 1999).

Oxidative stress is caused by an imbalance between the cell antioxidant mechanism and ROS production (Toledano et al., 2003). ROS includes different oxidation states of dioxygen (O_2_), such as singlet oxygen, superoxide anion (O_2_^−^), H_2_O_2_, and hydroxyl radical (OH •). These species are invariably produced in aerobic environments by different mechanisms, such as the “leakage” of electrons during biological oxidations, the action of flavin dehydrogenases, and the physical activation of oxygen molecules by radiation energy (Toledano et al., 2003; Bergamini et al., 2004).

It has been reported that visible light in *S. cerevisiae* alters its metabolism through changes in respiratory oscillation and the expression of the *YAP1* gene, which codes for yeast activator protein-1 and is related to oxidative stress (Robertson et al., 2013). Under this stress, the cell has different responses depending on how severe the exposure to ROS is. At low doses, cells would adapt, becoming more resistant to a subsequent lethal dose (Jamieson, 1992). At higher doses, the cell activates antioxidant defense mechanisms at the transcriptional level, mainly through transcription factors, such as the Yap1p mentioned above, Msn2p, and Msn4p (Jamieson, 1998; Gasch et al., 2000; Moradas-Ferreira and Costa, 2000), also causing a delay in cell division (Lee et al., 1996; Chiu et al., 2011). Even higher doses can cause the death of a part of the population initially due to apoptosis and finally in extreme doses due to necrosis (Zong, 2006; Perrone et al., 2008; Farrugia and Balzan, 2012).

Considering the importance of *B. bruxellensis* at the industrial level, it is interesting to research how this yeast responds to two stresses present in its natural habitat: *p*CA and light, allowing a deeper understanding of the metabolic ways of this spoilage yeast.

## Materials and Methods

### Microorganism

*B. bruxellensis* LAMAP2480 strain was obtained from the collection at the Laboratory of Applied Microbiology and Biotechnology of the University of Santiago de Chile. The strain was grown in SD minimal medium (2% w/v glucose and 6.7g/l yeast nitrogen base (YNB; Difco Laboratories, Detroit, United States) and kept in the dark until use).

### Growth Curves

*B. bruxellensis* LAMAP2480 strain was grown in 5ml of SD minimum medium (2% w/v glucose and 6.7g/l yeast nitrogen base (YNB; Difco Laboratories, Detroit, United States)) at 28°C until saturation (stationary phase). Then, 1×10^5^ cells/ml were inoculated in 200μl of the same medium in the absence and presence of 100mg/l of *p*CA (Sigma-Aldrich, Inc.; United States) in triplicate on a Cell Culture Plate (SPL Life Sciences, Korea). The microplates were incubated at 28°C in the dark and at different light intensities using white fluorescent lamps at a light intensity of 2,500 lux and 4,000 lux. Varying light intensities were provided by adjusting the light with the help of Lux meter UNI-T UT 382 USB (Dongguan, China). Absorbance measurements were made at 600nm for 10days in the Epoch^™^ equipment (BioTek, United States), coupled to the Gen5 program (BioTek, United States).

The specific growth rate was determined by the slope of the exponential growth phase according to the equation xt=x0+μt, where xt and x0 represent the biomass in optical density (OD) at time t (h) and *t*=0, respectively (Barata et al., 2008). The *lag* phase was determined as described by Buchanan and Cygnarowicz (1990). All experiments were performed in triplicate.

### RNA Extraction

*B. bruxellensis* LAMAP2480 strain was grown in SD minimal medium (2% w/v glucose and 6.7g/l yeast nitrogen base (YNB; Difco Laboratories, Detroit, United States)) at 28°C until saturation. 1×10^5^ cells/ml were inoculated in 100ml of medium in the absence and presence of 100mg/l of *p*CA (Sigma-Aldrich, Inc.; United States) at different light intensities (2,500 lux, 4,000 lux and in darkness). They were grown at 28°C until the end of *lag* phase (Godoy et al., 2016). Then, the culture was centrifuged at 1,370×*g* for 10min. The pellet was resuspended in 200μl of RNA Buffer (50mm Tris–HCl pH 7.4; 100mm NaCl; 10mm EDTA), and 400μl of acidic phenol and glass beads were added. Vortex 1min, then ice 1min, and repeat 3min in vortex. 200μl of RNA Buffer and 40μl of 10% SDS were added. It was stirred for 6min at 65°C. It was centrifuged at 16,000×*g* for 15min at 4°C, and the upper phase was collected. 400μl of acid phenol and 40μl of 3M sodium acetate were added, centrifuging at 16,000×*g* for 15min at 4°C to finally collect the upper phase. Subsequently, 1ml of cold 96% ethanol was added, and it was refrigerated for 2h at −80°C. It was centrifuged at 16,000×*g* for 10min at 4°C. Finally, the RNA Clean & Concentrator^™^ -5 protocol (Zymo Research, United States) was followed. RNA quantification was performed in the Epoch^™^ equipment (BioTek, United States). All experiments were performed in triplicate.

### Quantification of Relative Expression

The RQ1 RNase-Free DNase protocol (Promega, USA) was used for the RT-PCR. q-PCR was performed in qStepOnePlus Real-Time PCR System Thermal Cycling Block equipment (Thermo Fisher Scientific, United States) coupled to StepOne Software (v2.0; Applied Biosystems, United States). All the primers used were designed to amplify fragments between 100 and 300bp (Table 1). All reactions were performed in 20 uL according to the 5x HOT FIREpol EvaGreen qPCR Mix Plus (ROX) protocol (Solis Biodyne, Estonia). The program used was as follows: 15min at 95°C, 35 amplification cycles at 95°C for 15s, 60°C for 20s, and 72°C for 20s.

**Table 1:** List of primers used for RT-qPCR.

Primer (5′-3′)	Sequence	Tm (°C)
ACT1 F	GGT GAT GAC GCT CCA AGA	64
ACT1 R	TTG ACC CAT ACC GAC CAT AA	63
SOD1 F	GAG GGT AAC GAT CCA AA	58
SOD1 R	CAA AGA ACC AGC ATC AC	58
GCN4 F	CCA GGT GCT CTT ATC TC	58
GCN4 R	CTC AGT ATT CCT AGC TCT C	58
HSP12 F	AAA CCA GCC ATC GAA AC	60
HSP12 R	CTC AAA GAG AGG AAG ACA AG	59
ESBP6 F	CAC GCA TAC CCT TTA TC	57
ESBP6 R	GAG GAA CAA GCA AGA AG	57

The relative quantification of the expression of the candidate genes of *B. bruxellensis* was carried out using the mathematical method 2−ΔΔ𝐶𝑇 described by Livak and Schmittgen (2001), using actin 1 as the housekeeping gene. All experiments were performed in triplicate.

### Statistical Analysis

The statistical analyses were carried out using ANOVA, and the mean values of the experiments were compared using the LSD test. The treatments were considered significant when the values of *p* ≤0.05. The analyses were done using Statgraphics Plus, version 5.1 (StatPoint Technologies, United States).

## Results

### Growth of *B. bruxellensis* in the Presence of Light and *p*-Coumaric Acid

*B. bruxellensis* is the main contaminating yeast in the wine industry since it causes defects in the organoleptic properties of wine. Although SO_2_ is the compound that manages to control the growth of this yeast, it has been described that this compound can cause allergy problems in consumers, hence the interest in finding new control methods. *B. bruxellensis* can grow in various environmental stress conditions, including low nitrogen, low vitamin, high SO_2_ doses, low or high oxygen concentration, and high ethanol concentration. Considering this, innovative methods have been developed to reduce their contamination in wines, as are high hydrostatical pressure (HHP), pulse electric fields (PEF), ultrasound, UV light, and microwaves, among others (Pinto et al., 2020). These technologies are being studied to can be applied in the industry.

**Figure 1:**
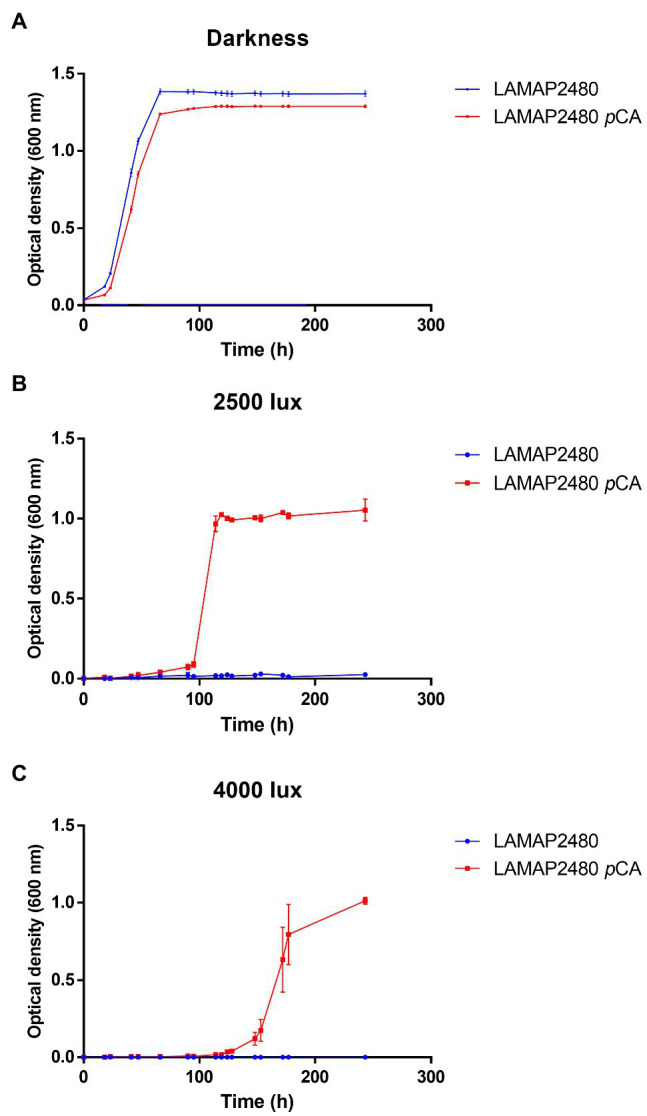
Growth curves of *Brettanomyces bruxellensis* LAMAP2480 in the absence and presence of 100mg/l of *p*CA. In **(A)** darkness. In **(B)** exposed to 2,500 lux and **(C)** exposed to 4,000 lux. The experiments were done in triplicate.

On the other hand, studies carried out in *S. cerevisiae* have shown that both the presence of hydroxycinnamic acids in the culture medium and light would affect its growth (Ułaszewski et al., 1979; Baranowski et al., 1980; Shu et al., 2009; Robertson et al., 2013). We evaluated how *B. bruxellensis* LAMAP2480 responds to these two factors (*p*CA and light). Growth curves were made in the absence and presence of *p*CA, either in darkness or exposed to 2,500 and 4,000 lux, calculating their kinetic parameters (Figure 1A; Table 2). It is possible to observe that when the yeast was grown in darkness, the duration of the *lag* phase was 26.25h in the absence and in the presence of *p*CA (Table 2). This parameter, such as specific growth rate and generation time, did not show significant differences when comparing yeast behavior in the presence or absence of *p*CA.

**Table 2:** Kinetic parameters of growth *B*. *bruxelllensis* LAMAP2480 under different conditions.

Condition	Specific growth rate μmax (h^−1^)	*lag* phase (h)	Generation time [Tg (h)]
Darkness	0.0274±0.0004^b^	26.25±0.00^a^	25.27±0.32^b^
Darkness *p*CA	0.0264±0.0001^b^	26.25±0.00^a^	26.29±0.11^b^
2,500lx	NG^*^	NG^*^	NG^*^
2,500lx *p*CA	0.0462±0.0017^a^	98.5±0.00^b^	15.01±0.57^a^
4,000lx	NG^*^	NG^*^	NG^*^
4,000lx *p*CA	0.0253±0.0058^b^	162.5±8.49^c^	28.14±6.45^b^

*Mean±SD (n=3). Different letters after means represent significant differences between conditions (p≤0.05)*.

* *NG: no growth. The experiments were done in triplicate*.

In addition, when *B. bruxellensis* was exposed to a constant light intensity of 2,500 lux (Figure 1B; Table 2) in the presence of *p*CA, it was observed that the duration of the *lag* phase increased 3.75 times compared to control sample (darkness+ *p*CA) and its specific growth rate increased 1.75 times. The growth efficiency (calculated as area under the curve, considering 100% darkness + *p*CA) decreased by 55.8%.

When the yeast was exposed to 4,000 lux (Figure 1C; Table 2), there was an increase in the duration of the *lag* phase of 6.19 times compared to the sample control (darkness + *p*CA). The specific growth rate and generation time parameter did not show statistically significant differences with respect to the control. Additionally, the growth efficiency was 28.6%, 3.5 times lower compared to the control.

Also, by comparing the growth of the cells to 2,500 lux and 4,000 lux, it was observed that the *lag* phase was lower to 2,500 lux (Figures 1B,C).

Furthermore, it was observed that yeast growth was negatively affected when it was exposed to both light intensities and in the absence of *p*CA, where no growth was detected.

### Relative Gene Expression

**Figure 2:**
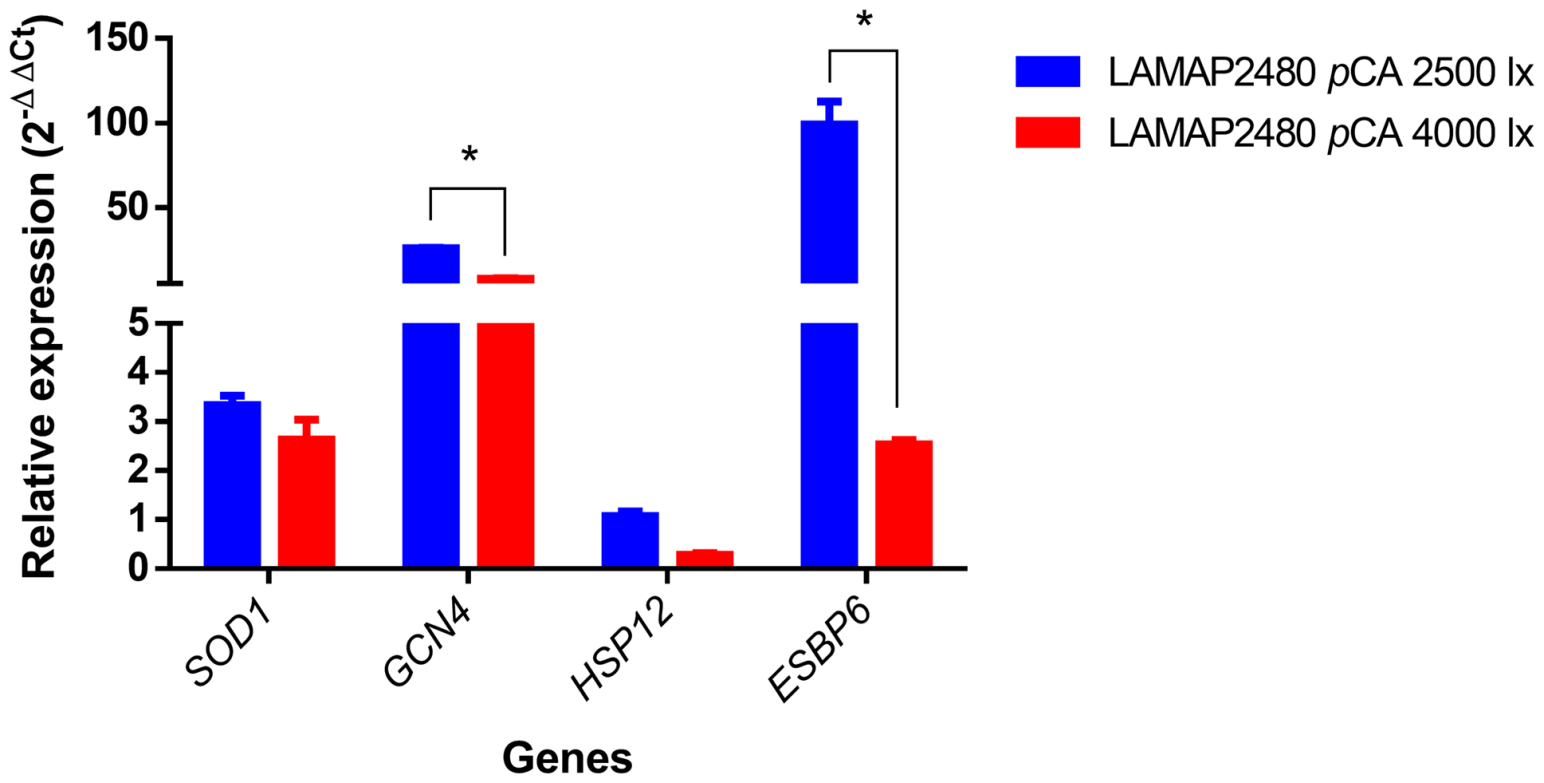
Relative expression of genes related to oxidative stress during the *lag* phase of B*. bruxellensis* LAMAP2480 in the presence of *p*CA. Data were statistically analyzed using least significant difference (LSD) statistical test with a 95% confidence level. Asterisks represent significance (^*^p<0.05).

Light would induce gene expression related to oxidative stress (Toledano et al., 2003; Perrone et al., 2008). To evaluate the response of genes associated with this type of stress in *B. bruxellensis* LAMAP2480, different light intensities were tested. The relative expression at the end of the *lag* phase of some genes previously associated with oxidative stress in *S. cerevisiae* was quantified in *B. bruxellensis* in this study (Figure 2).

These results indicated that *SOD1*, *GCN4*, and *ESBP6* genes were overexpressed (ER ⩾ 2) at 2,500 and 4,000 lux. Particularly, the *GCN4* and *ESBP6* genes expression was 3.3 and 39.6 times higher respectively, compared to the 4,000 lux condition, being statistically significant.

For the *HSP12* gene, no overexpression was observed in any condition.

## Discussion

The study of the growth control of *B. bruxellensis* is very important in winemaking, considering the need to reduce the use of SO_2_. This yeast has shown great resistance to different stress conditions during its growth in wine, being able to form spores (*Dekkera* species teleomorph) to survive. *B. bruxellensis* is resistant to different enological factors as high ethanol concentration, low pH, low oxygen transfer rate, and residual ammonium scarcity (Curtin et al., 2015; Pinto et al., 2020). Hence, it the importance of looking for new control methods to avoid contamination in winemaking.

Folch-Mallol et al. (2004) reported a mechanism called cross-protection in *S. cerevisiae*. This consists of adapting the microorganisms to a specific type of stress, which would allow it to resist other stress factors, although these are lethal in the absence of a previous induction. The molecular base would be to activate different stress genes involved in adaptive responses (Brown et al., 2014; Święciło, 2016). In this sense, the light stress to which *B. bruxellensis* LAMAP2480 was exposed in the absence of *p*CA affected yeast growth; however, the presence of another stressor, such as *p*CA, allowed *B. bruxellensis* LAMAP2480 to adapt to light stress, observing an increase in the expression of genes related to stress to oxidative, suggesting a possible stress-protective effect by *p*CA at cellular level.

Various environmental stress conditions, including high light intensities, extreme temperatures, and metals, among others, can induce oxidative stress and accelerated formation of ROS. In general, it has been described that visible light would produce ROS by endogenous photosensitizers, such as flavins and porphyrins (Molin et al., 2020). In this respect, hydroxycinnamic acids, such as *p*CA, and flavonoids, normally present in plants and fruits, exhibit radical scavenging ability (Scandalios, 2005; Yingbin et al., 2019).

In agreement with our results, Bayliak et al. (2016) evaluated the effect of quercetin, one flavonoid, on stress resistance of exponentially growing *S. cerevisiae* cell exposure to hydrogen peroxide, copper ions, and heat shock. Quercetin increased stress resistance in the yeast *S. cerevisiae via* antioxidants related at high concentrations providing partial protection to proteins against ROS-induced modification under heat shock and oxidative stress exposure.

There are no studies of the effect of light on the growth of *B. bruxellensis*, but few have been carried out on *S. cerevisiae* (Woodward et al., 1978; Ułaszewski et al., 1979; Edmunds, 1980; Robertson et al., 2013). The same occurs with studies at the genetic level on changes in gene expression in these yeasts when exposed to visible light.

Woodward et al. (1978) observed that at 1250 lux, the growth of *S. cerevisiae* is not affected; however, when increasing the intensity of the light (over 1,250 lux), the generation time of the yeast increases progressively. Studies carried out in *S. cerevisiae* indicate that this is due to the inhibition of the transport of sugars and amino acids. Furthermore, it was described that increasing growth temperature and light intensity also has a negative effect on yeast growth. Robertson et al. (2013) observed that *S. cerevisiae* expresses yeast activator protein-1 gene (*YAP1*) related to ROS response when exposed to visible light. This gene regulates several genes with antioxidant functions, such as thioredoxin reductase (*TTR1*), cytosolic thioredoxin (TRX2), and cytochrome-c peroxidase (*CCP1*). Yap1p would play an important role in the yeast’s ability to tolerate the harmful effects of visible light. These same authors showed that in those cells with a deficiency in the ROS response, light would have a negative effect on their growth.

Like *YAP1*, *GCN4* is part of the b-ZIP family of transcriptional factors (Rodrigues-Pousada et al., 2010) and has been shown to protect the cell under conditions of oxidative stress (Jamieson, 1998; Folch-Mallol et al., 2004; Mascarenhas et al., 2008). In *S. cerevisiae*, it has been reported that mutants lacking the *GCN4* gene do not grow when exposed to different concentrations of H_2_O_2_. Our results show overexpression of *GCN4* gene in both light intensities, which could be related to the antioxidant response induced by the presence of *p*CA in the medium.

*SOD1* encodes a cytosolic copper-zinc superoxide dismutase which catalyzes the dismutation of the superoxide radical (O_2_^–^) to oxygen (O_2_) and H_2_O_2_ (Steinman, 1980). de Nobel et al. (2001) reported a comparative analysis between the proteome and transcriptome of *S. cerevisiae* when exposed to sorbic acid, which is a weak acid as *p*CA is. The results indicated that *SOD1* is overexpressed in response to sorbic acid in *S. cerevisiae*, suggesting that it is part of the resistance mechanism to this acid. Some microorganisms can adapt quickly to a second stress type, having been previously exposed to another stress. For example, in *Candida albicans*, oxidative stress induces heat shock genes regulation, and *S. cerevisiae* has protection against oxidative stress if cells have been exposed to a mild heat shock. This protection stress would have unexpected responses to classical regulatory pathways to a specific stress due to combinatorial cross-talk (Brown et al., 2014).

Molin et al. (2020) have concluded that the protein kinase A plays a key role in yeast growth in the presence of light, which would be a circadian mechanism that is somehow conserved in yeast and mammals. These authors demonstrated that the presence of light reduces protein kinase A activity, which would be vital for cells when grown in the presence of light since mutants that were sensitive to light had a protein kinase A activity well above those of normal levels.

Sugiyama et al. (2016) reported the overexpression of *ESBP6* gene in the presence of lactic acid. Through gene disruption and overexpression experiments, *ESBP6* gene was shown to be involved in response to lactic acid adaptation in *S. cerevisiae*, although it does not appear to be involved in the transport of monocarboxylic acids. The overexpression of this gene suggests that it plays a fundamental role in the adaptation of *S. cerevisiae* to lactic acid. *ESBP6* interacts with various proteins in response to stress (Hsp70, Hsp82, Hsp90, Ssa1, and Ssa2). These chaperones allow the refolding of denatured proteins, stabilize the interaction of proteins or transport, and degrade damaged proteins, which is crucial in adapting to weak acids since this causes an increase in intracellular pH, affecting the structure and function of the yeast proteins. In addition, Pereira et al. (2020) demonstrated that overexpression of *ESBP6* gene generated greater resistance to aromatic acids, including *p*CA, reducing the stress level in the cell by promoting biomass yield. Our results suggest that *p*CA-induced overexpression of *ESBP6* has a protective effect against light stress, allowing cell growth.

Cross-stress can show a low specify in defense/reparation mechanism, allowing alternatives mechanism with a similar biochemical response to assure important functions of a cell as a response to different types of stress can be translated into a stress adaptation highly relevant to the natural environment (Święciło, 2016).

It has been described that the incidence of light on yeast growth would depend on the initial concentration of the culture. It is possible that by increasing the cell biomass, there is protection of the external cells over the internal cells of the culture. The outer cells would absorb light energy, preventing the inner cells from being affected by the toxic effects of light, allowing their normal division (Molin et al., 2020). Also, we think the phase of growth is important. In the case of saturation phase, some of the principal structures to inhibit the effects of light should be actives, as are the cytochromes (Ułaszewski et al., 1979), or in this stage, the cellular wall should be totally formed, transformed into a barrier for the entry of light to the interior of the cell. Our experiments were carried out with the same initial concentration of cells, but it is an interesting point to consider for future trials.

Different environmental factors can activate the response to oxidative stress in microorganisms. Among them, we have the presence of hydroxycinnamic acids (a natural compound) and sunlight. In addition, it has been described that if a microorganism is exposed to more than one stress factor, it will acquire an overprotective quality. That is, the cell could trigger more than one defense response in such a way as to allow it to adapt to grow in the presence of various stressors. To our knowledge, our study is the first report on the effect of light on *B. bruxellensis* growth.

Our results showed that, in the case of *B. bruxellensis*, when exposed to a concentration of 100mg/l of *p*CA and in the absence of light, the duration of the *lag* phase slightly increased, since after adapting to the culture medium, its growth reached similar optical density values as the control sample (absence of *p*CA). It was also observed that when yeast was exposed to *p*CA and light (2,500 and 4,000 lux), the duration of the *lag* phase was statistically longer compared to when the yeast is grown with *p*CA in the absence of light. This suggests that the response of *B. bruxellensis* LAMAP2480 would be related to cross-protection when exposed to both stressors. However, other effects of *p*CA and light on the cells should not be discarded.

Furthermore, expression levels of the genes indicated overexpression of the *SOD1, GCN4*, and *ESBP6* genes for both light intensities in the presence of *p*CA, suggesting that the presence of *p*CA stimulates an antioxidant response allowing the growth of *B. bruxellensis* exposed at 2,500 and 4,000 lux. Although the effect of light causes an increase in the duration of *lag* phase, *B. bruxellensis* LAMAP2480 can adapt and, despite having a decrease in growth efficiency, can grow to a high concentration of cells.

## Data Availability Statement

The original contributions presented in the study are included in the article/supplementary material, further inquiries can be directed to the corresponding authors.

## Author Contributions

LG, DC, and MG: conceptualization and writing – preparation of original draft, review, and editing. LG and DC: formal analysis and methodology. MG: funding acquisition. MS: supervision. All authors have read and agreed to the published version of the manuscript.

## Funding

This research was funded by ANID/CONICYT FONDECYT Iniciación 11180979 and Postdoct DICYT 081871GM POSTDOC Universidad de Santiago de Chile. Millennium Nucleus for Fungal Integrative and Synthetic Biology (NC120043) and Dicyt-USACH.

## Conflict of Interest

The authors declare that the research was conducted in the absence of any commercial or financial relationships that could be construed as a potential conflict of interest.

## Publisher’s Note

All claims expressed in this article are solely those of the authors and do not necessarily represent those of their affiliated organizations, or those of the publisher, the editors and the reviewers. Any product that may be evaluated in this article, or claim that may be made by its manufacturer, is not guaranteed or endorsed by the publisher.
